# The role of aggression and maladjustment in the teacher-student relationship on burnout in secondary school teachers

**DOI:** 10.3389/fpsyg.2022.1059899

**Published:** 2022-11-24

**Authors:** Barbara Masluk, Santiago Gascón-Santos, Bárbara Oliván-Blázquez, Cruz Bartolomé-Moreno, Agustín Albesa, Marta Alda, Rosa Magallón-Botaya

**Affiliations:** ^1^Department of Psychology and Sociology, University of Zaragoza, Zaragoza, Spain; ^2^Research Network on Preventive Activities and Health Promotion (RICAPPS), Barcelona, Spain; ^3^Aragón Health Service, Government of Aragón, Zaragoza, Spain; ^4^Institute of Health Research of Aragón (IIS Aragón), Miguel Servet University Hospital, Zaragoza, Spain

**Keywords:** violence, adjustment in teacher-student relationship, burnout, secondary school teachers, compulsory education

## Abstract

**Introduction:**

Multiple studies have examined the individual and socio-demographic variables that can contribute to the development of burnout in teachers. Although the evidence supports that this syndrome is generated through the interaction between the aspects of the organization and those of the person, little attention has been spent on the impact of the teacher-student relationship adjustment and, especially, on the role of violence exercised by students or their families toward secondary school teachers, who seem to be more vulnerable than teaching professionals in general.

**Objective:**

To analyze the role of the possible mismatch in the student-teacher relationship, as well as, the physical and verbal violence toward teachers from pupils or their parents, on the professional wear of high school educators.

**Materials and methods:**

A cross-sectional study was carried out on a teacher sample (*n* = 677) in Aragón, Spain, through a questionnaire with socio-demographic data; the “Maslach Burnout Inventory” (MBI), “Areas of Worklife Scale,” the “Fears and Rejection in Education Questionnaire” (FREQ), and a list of the possible aggressions received in the development of the teaching activity.

**Results:**

While 3.8% of teachers have been a victim of physical attacks, 34.9% have suffered verbal abuse at least once. Although physical violence is extremely rare (and low intensity), verbal victimization or threats are associated with burnout in a highly significant manner, which confirms previous findings about school violence and burnout. Also, FEAR and REJECTION dimensions, defined as discomfort, tension, anxiety, and pressure caused by pupils, which contributed considerably on two dimensions of burnout (emotional exhaustion and cynicism). Different covariates such as maladjustment in the teacher-pupil relationship, violence experienced at work, and complaints received explain the 56.4% variance of exhaustion, 48.8% variance in cynicism, and 35.5% for efficacy.

**Conclusion:**

Very different variables can contribute to the development of burnout syndrome, both personal, and organizational variables. Therefore, when designing prevention programs in each work environment, the possible areas of risk and the interactions between them must be considered.

## Introduction

Education professionals are one of the sectors most affected by work stress (von der Embse et al., 2019). Stress in teachers has also been widely addressed and recognized as a major problem worldwide (Loonstra et al., 2009; Van Droogenbroeck and Spruyt, 2015. Teachers have to comply with many heterogeneous responsibilities in their work (daily routines, student performance, and satisfying parent demands), which define teaching as a profession of special risk (von der Embse et al., 2019). This and other facts, such as little recognition, or lack of institutional support, is reducing the number of highly capable workers in the education sector (Donahue-Keegan et al., 2019).

Burnout syndrome is described as a behavioral manifestation of work stress, always in the context of an organization where “emotional exhaustion, depersonalization (or cynicism), and low personal fulfillment can occur frequently among individuals whose work involves care or assistance to people” (Maslach et al., 1997). Work stress and burnout are related not only to sick leave and drop-out rates, but also to general and mental health problems (Toppinen-Tanner et al., 2009). According to several authors, low control, high demands, and imbalance of efforts have been shown to play an important role in predicting risk of premature death (Vahtera et al., 2004).

There has been controversy about the possible absence of pathognomonic signs of its own when considering the syndrome as a disorder and, frequently, attempts have been made to reduce it to a kind of depression caused by work (Bianchi et al., 2013). Maybe for this reason, it is not considered in the DSM-V classification as an entity of its own (American Psychiatric Association, 2013), although it could be included under the category of “adaptive disorder,” coinciding with the development of emotional or behavioral symptoms in response to an identifiable psychosocial stressor (Kakiashvili et al., 2013). But the syndrome is somewhat different and, in addition to the characteristic signs and symptoms of depression, other indicators are present. Emotional exhaustion is the central feature, but the syndrome is accompanied by feelings of depersonalization and also by an increasing lack of professional fulfillment from work (Maslach et al., 1997). On the other hand, affected people do not always respond to treatment with antidepressant drugs, they usually show improvement if they are away from work for a while and; when they run out of their time limit due to sick leave, some decide not to return to their job, even they will refuse to accept another job within their profession (Kowalczuk et al., 2020).

Given the overwhelming evidence, the World Health Organization recognized this syndrome as an occupational condition that affects millions of workers around the world (Reed et al., 2019).

In recent decades, there have been many studies that have tried to explain burnout, either through socio-demographic variables, or due to causes related to personality traits, or due to the presence or absence of abilities or skills, such as resilience, proactive strategies (Pietarinen et al., 2013), emotional intelligence (Ju et al., 2015), and satisfaction and motivation (Skaalvik and Skaalvik, 2011).

Although many authors point out that gender, seniority, and level of education imparted can predict burnout (Burke and Greenglass, 1995), except for the moderating effect of social support, there are no conclusive results whit respect to socio-demographic aspects. According to Ahola et al. (2006), living in a stable relationship, seems to be a factor which prevents depersonalization (or cynicism). But it has also been found that not all workers in the same company feel equally affected, and different subtypes of burnout have even been described–“frenetic,” “under-challenged,” and “worn-out”–depending on the type of personal characteristics (Montero-Marín et al., 2013).

Research on this syndrome has repeatedly confirmed that one should not focus on individual variables, and that the phenomenon is generated through complex interactions between the variables of the organization and those of each person (Schaufeli et al., 2009). Based on the results of several surveys on work-related stress, Maslach and Leiter (2008) also offered a model based on the variables or “Areas of Work Life,” which could contribute to burnout experience. The proposed areas are: workload, control, rewards, fairness, values, and community. Of these, the community area refers to the relationships between employees and their bosses, colleagues and the people they serve.

Personal relationships, especially those based on the demands of clients, patients, and students, are of great importance in the accumulation of stress and the development of burnout (Bakker et al., 2000). In this type of relationship, adequate emotional management plays a crucial role, often producing a containment or repression of emotions (Zhao and Ding, 2019). Professionals can live daily with emotions such as fear, anxiety, even situations of violence, which can contribute to emotional erosion (Gascón et al., 2009).

There are many studies on the climate in class, but not so many on adjustment of the teacher-student relationship has received less attention (Corbin et al., 2019; Teuber et al., 2021). The topic of the relationship between teacher and student was addressed by Claessens et al. (2017), whose results conclude that teachers defined the quality of the relationship mostly by the level of communication (friendly vs. hostile), instead of by the level of action (in control vs. powerless).

A high level of student demands can predict teacher burnout symptoms (Teuber et al., 2021) and it has been shown that students’ conflictive relationships with teachers are related to higher emotional exhaustion and lower personal fulfillment (Corbin et al., 2019). On the other hand, the absence of a positive atmosphere in class increases the possibility of burnout and, as a consequence, will diminish the affective commitment with the organization (Hakanen et al., 2006); feelings of justice at work, also, provide a buffer against burnout (Riolli and Savicki, 2006).

Until recently, student aggression toward teachers have not been sufficiently studied (Tiesman et al., 2014; Berg and Cornell, 2016), and has focused mainly on aggression among pupils. The conflict between teachers and students and, more recently, aggression in education, have been the object of analysis in relation to professional burnout (Krause and Smith, 2022). Aloe et al. (2014) made a contribution to the literature by conducting a multivariate meta-analysis aimed at exploring the relationship between student misbehavior and the dimensions of burnout. Episodes of violence and the possibility of suffering continuous aggression have been shown as explanatory variables of burnout in other professions due to their role in the development of the syndrome (Gascón et al., 2013a).

Smith and Smith (2006) showed that the threat of violence in schools was the main contributing factor teachers faced when teaching at an urban school. In U.S., Tiesman et al. (2014) found that teachers who work in an urban school were almost four times more likely to have been physically assaulted than those who work in rural schools. Physical aggression had a significant impact on the job satisfaction of education workers and on health-related quality of life. Having experienced at least one physical aggression during one course increased the probability of seeing work as stressful up to twice as much, and entailed 2.4 more probability of not being satisfied with their work. In addition, those who had been physically assaulted were almost 11 times more likely to present intention to quit their jobs (Krause and Smith, 2022).

The reality in Europe is not different, especially with regard to conflict in compulsory secondary education. In Spain, secondary education begins at age 12 and lasts until age 18, and it is compulsory until the age of 16. Teachers enter the state education system by passing a competitive entrance exam; however, passing the exam does not initially ensure tenure and job stability, as new teachers have to take up posts far from their place of residence or in remote areas, until they have built up the seniority necessary to choose a location more suited to them (Botía, 2004). The hardness of the access system, the conditions of the first decades, deficits in promotion development, low wages, the loss of status, and the difficulties involved in working with conflictive and violent students, contributes to a gradual lack of motivation and the wear and tear of these professionals (Gadella Kamstra, 2020).

The purpose of this study is to examine the contribution of work-related factors such as adjustment in the teacher-pupil relationship, threats, complaints, and aggressions on core dimensions of teacher burnout in Spain.

## Materials and methods

### Participants

A cross-sectional, descriptive study was carried out in Aragón (Spain) in 2019. Participants included 677 teachers from a total of 34 schools in the state and private sectors. Previously, a pilot study was developed with 148 participants with the aim of validating the FREQ questionnaire.

The following inclusion criteria were considered: that the teacher had been working over the last 12 months and that they agreed to answer the questions by signing the informed consent. During the fieldwork, we were able to count on the support of facilitators, i.e., teachers who were in charge of ensuring compliance with eligibility criteria and of delivering and collecting the sealed surveys to ensure the confidentiality of the data.

The total number of participants in state schools was 587 (86.7%) and 90 in private schools (13.3%). Based on the data provided by the Institute of Statistics of the Government of Aragon, in the year 2019 there have been 4,144 teachers of secondary education in total; 3,493 in public centers and 621 in private centers. The sex distribution data is representative with respect to official statistical data among secondary school teachers: 59.7% were women and 40.3% were men (Instituto Aragonés de Estadística, 2021).

### Measurement instruments

– *Socio-demographic variables* were evaluated through a questionnaire which included: gender; age; family situation; years of experience and type of position (tenured position or interim teacher). Other variables collected were the intention to give up the profession, contract type (public official vs. resident vs. temporary worker), type of school (public vs. private), and environment (urban vs. rural).
– The three burnout variables were measured through the questionnaire. *The Maslach Burnout Inventory-General Survey (MBI-GS)* (Maslach et al., 1997), validated for the Spanish population (Salanova and Schaufeli, 2000), and provided the information related to: exhaustion; cynicism; and personal accomplishment. The concept of “cynicism” in Spanish has a confusing translation, since it refers to a negative moral trait, while the dimension of the questionnaire describes an experience of skepticism, withdrawal, or psychological distancing. In any case, it was decided to keep the word as it appears in the MBI-GS. The 16 items must be answered on a Likert scale that ranges from 0 (never) to 6 (always). To obtain the scores of each scale, it is necessary to add scores obtained in each item of the scale and divide the result by the number of items of each scale. The indices and reliability of the three scales are good with Cronbach’s α > 0.70. When interpreting the results, the burnout pattern translates into high exhaustion, cynicism, and low performance.
– Areas of Worklife Scale (Leiter and Maslach, 2003). It consists of 29 items that evaluate the degree of fit or mismatch that the professional perceives about the variables of their work environment: workload, control over the tasks, intrinsic rewards at work, community, fairness, and values (concordance between own values and those of the organization). The scale considers the degree to which the subject agrees or disagrees with statements related to the areas of their work life (e.g., “I don’t have time to do the work that must be done”). The Spanish version gained a Cronbach’s Alpha ranging from 0.75 to 0.88: Workload = 0.80; Control = 0.87; Reward = 0.84; Community = 0.75; Fairness = 0.88; and Values = 0.85 (Gascón et al., 2013b).
– The measure of imbalance in student-teacher relationships was obtained by the self-made survey FREQ. The test contained 14 items, and participants were asked to indicate how much they felt secure in their student-teacher relationship on a seven Likert scale ranging from 0 (*never*) to 6 (*on a daily basis*). An example of a question is: *I feel pressured by students/parents to change my decisions*. The FREQ was developed using qualitative methodology (Rahman, 2020) using a sample of 29 teachers, divided into four groups. All the areas considered important and essential were listed for the questionnaire’s content validity. Fears, stress sources, situations which cause rejection in the profession were openly discussed. The lists obtained in each group were unified and handed back to the teams for them to order those situations from most to least importance. The provisional list obtained was discussed by a team of nine teachers from four groups. The definitive questionnaire was completed for its validation, to secondary teachers in three provinces of Aragon (*n* = 148; 56.7 women; mean age = 44.3; SD = 7.51). Statistical analysis confirms two dimensions, which contributed to the deterioration of the relationship between teachers and students, one called “fears” (fear of disturbing or making mistakes and its consequences) and another which was called “rejection” (annoyance and displeasure). The questionnaire showed an α indices 0.835. A more detailed description of the psychometric analysis can be seen in the Section “Results.”
– *Questionnaire on violence:* a list was used describing the types of violence that the California Occupational Safety and Health Administration (California Division of Occupational Safety and Health Administration., 1995) defines as Type II–exercised by users, patients or students–, without reflecting the aggressions that are not related to the work environment or those carried out by the staff. Each item describes a type of aggression: physical, verbal threat, threatening behavior, and verbal abuse (Winstanley and Whittington, 2004). Made up of four items, the participant is asked to respond on a five-point Likert scale between 0 *never* and 4 *on more than 5 occasions*. An example of a question is: *I have received threats (verbal threats, threatening behavior or coercion).* In addition, the participants reported “yes” or “no” to the questions about whether they had received specific training in relation to aggression, if they felt supported by the Administration/Management in cases of violence, and if they have received claim or complaints from students or their families.

## Results

The Statistical Package SPSS 27.0 (IBM Corp., Armonk, NY, United States, Released 2012). IBM SPSS Statistics for Windows has been used to perform all the statistical analyses of the study.

### Validation of the Questionnaire of Fears and Rejection in Education

The psychometric properties of the FREQ were analyzed, obtaining values of α = 0.908.

This study validated a two-factor CFA model, using principal components analysis with VARIMAX rotation were obtained. In addition, a confirmatory factor analysis (CFA) was used to test whether the factorial structure was consistent. The CFA produced a fit [χ^2^ (91) = 957,937, *p* < 0.001; χ^2^/df = 0.799; CFI = 0.756; RMSEA = 0.00] much better than a One Factor Model that assigned all items to a single factor. The questionnaire showed a structure of two factors (Table 1) that explain the 67.17% of common variance of the variable mismatch in the teacher-student relationship. The first factor, FEARS (fear of error and its possible consequences), accounts for 36.81% of the total variance and the second factor, REJECTION (discomfort, tension, and pressure caused by pupils), accounts for 29.36% of the variance.

**TABLE 1:** Principal components factor analysis (PCFA) of Fears and Rejection in Education Questionnaire (FREQ).

	Item			
1	I am afraid that my words may upset the students	FEARS	0.620	0.362
2	I am afraid of making mistakes and their possible consequences		0.708	0.209
3	I am afraid to ask students about some questions		0.675	0.158
4	I am afraid of being humiliated or attacked		0.867	0.121
5	I feel insecure when making decisions that affect students		0.641	0.339
6	I fear being accused of negligence		0.798	0.079
7	I feel helpless before my limits or those of the educational system		0.703	0.168

8	In general, students cause me anxiety and/or rejection	REJECTION	0.040	0.826
9	Some students provoke hostile behavior toward me		0.101	0.844
10	Trivial or unnecessary consultations or tutorials annoy me		0.325	0.671
11	I feel invaded by the demands of the students		0.351	0.594
12	I feel pressure from students/parents to change my decisions		0.225	0.689
13	I can’t stand parent/student complaints about my work		0.169	0.641
14	I do not accept complaints about the educational system		0.202	0.684

Extraction method: principal component analysis. Rotation method: Varimax with Kaiser normalization.

The rotation converged in three iterations.

### Data analysis

A descriptive analysis of the burnout dimensions (Table 2) was carried out to compare the means of the sample and the normative measures for MBI. Finally, the calculation of the likelihood of belonging to the burnout group was carried out according to the variables of interest using the lineal regression models LRM, performing analyzes for each variable separately in order to evaluate the unadjusted association with burnout (bivariate analysis).

**TABLE 2:** Means and normative means (Maslach et al., 1997) of the three dimensions of burnout in the sample.

	*N*	Mean	M	DT	Min.	Max.
Emotional Exhaustion	677	1.50	2.12	1.04	0.00	5.60
Efficacy	677	2.52	4.45	0.81	0.17	5.00
Cynicism	677	1.30	1.50	1.01	0.00	5.80

M, normative mean.

The variable status was divided into two groups: (1) in stable relationship or (2) divorced, single, or widowed. In order to proceed with the statistical analysis, some of the variables have been converted into dummy variables. For socio-demographic variables, the people in charge variable was divided into two categories: no dependents and with dependents. The socio-professional variables were introduced into the analysis as dummy variables as follows: years of experience [previously divided in (1) between 1 and 2 years; (2) between 3 and 6 years; (3) between 6 and 12, and (4) 6-over 12 years]; type of school (state or private); environment (urban or rural).

The work-related factors were: aggression: divided into physical aggression, verbal aggression (insults or denigration), and threats. Adjustment in the teacher-pupil relationship (FREQ), defined as the aspects that cause fear and rejection in the teaching relationship was also included, and having received complaints from students or their families.

### Description of the sample

The sample consisted of 677 subjects, 51.8% women and 48.2% men, with an average age of 44.6 years and a standard deviation (DT) of 9.5 years. In terms of marital status, 486 (71.8%) had a stable partner, while 163 (24.1%) had no partners. It was observed that 389 (66.3%) of the participants had children. 332 participants (49%) had dependents in their care. In regards to professional status, 453 (66.9%) were civil servants, which implies that 146 participants (21.6%) were interims and 57 of the participants (8.4%) were employed in private schools. Of the total sample, 62 (9.2%) were fully convinced that they would leave the profession in the near future. In terms of perceived health, 111 of the workers (16.4%) considered their health to be excellent, 482 (71.2%) workers rated their health as good, 72 (10.6%) reported having below average health and only three workers (0.4%) considered that their health was poor.

### Means and normative means

The results obtained in the sample are located in the scales below. The normative means that the authors (Maslach et al., 1997) have taken into account the measures used in different countries and different professions, while observing the largest difference in the efficacy scale, followed by emotional exhaustion. The cynicism scale had a score of 0.20 below the normative mean. A description of the burnout dimensions was performed according to normative data for the correction of MBI-GS scores in the Spanish population (Bresó et al., 2007).

It can be observed that the mean for the emotional exhaustion dimension according to normative data is at the medium-low level, the level of cynicism is at the medium-high level and, finally, the level of personal fulfillment is very low (see Table 3).

**TABLE 3:** Normative data for the correction of burnout dimension scores.

		Exhaustion	Cynicism	Efficacy
Very low	<5%	<0.4	<0.2	<2.83
Low	5–25%	0.5–1.2	0.3–0.5	2.83–3.83
Medium to low	25–50%	1.3–2	0.6–1.24	3.84–4.5
Medium to high	50–75%	2.1–2.8	1.25–2.25	4.51–5.16
High	75–95%	2.9–4.5	2.26–4	5.17–5.83
Very high	>95%	>4.5	>4	>5.83

### Correlational analysis

A Spearman Rho correlation was performed. The results show that all of the significant correlations between the study variables were in the hypothesized direction.

The demographic variables–age and partner–and the socio-labor variables–time in education, employment status (public official, interim, or contracted), type of center (public, concerted, or private)–showed statistically significant correlations with the dimension of cynicism, but not with the dimensions of exhaustion or efficacy (Table 4).

**TABLE 4:** Spearman correlation coefficient matrix for socio-demographic, work-related, and burnout dimensions.

	Exhaustion	Cynicism	Efficacy
Sex	–0.001	–0.120	0.154
Age	0.155	0.246**	0.109
Partner	–0.062	0.337**	0.070
Time in education	0.052	0.179*	–0.095
Status (hired/civil servant)	–0.178	−0.348**	0.113
Center (publ., concer., private)	–0.193	–0.117	0.153

**The correlation is significant at the 0.01 level (bilateral).

*The correlation is significant at the 0.05 level (bilateral).

As expected, the six areas of work life (workload, control, rewards, community, fair, and values) obtained statistically significant correlations with practically all the dimensions of burnout (Table 5), as well as the two dimensions of maladjustment in the teaching relationship–fears and rejection–, of which the FREAQ questionnaire reports.

**TABLE 5:** Spearman correlation coefficient matrix for areas of work-life, variables related to the mismatch in the teaching relationship, and burnout dimensions.

	Exhaustion	Cynicism	Efficacy
Workload	−0.408**	−0.291**	0.177*
Control	−0.287**	−0.309**	0.253**
Rewards	−0.418**	−0.391**	0.428**
Community	−0.322**	−0.198*	0.123
Fair	−0.327**	–0.141	0.234**
Values	−0.428**	−0.288**	0.265**
Fears	0.463**	0.390**	–0.126
Rejection	0.521**	0.428**	−0.217*
Physical assaults	0.038	0.052	−0.273**
Insults	0.350**	0.210**	−0.164*
Threats	0.285**	0.148**	–0.075
Complaints	0.173	0.205*	–0.180
Claims	0.277**	0.369**	–0.137

**The correlation is significant at the 0.01 level (bilateral).

*The correlation is significant at the 0.05 level (bilateral).

The physical aggressions (3.8% had suffered) was of low intensity and only found a statistically significant correlation with the dimension of efficacy. However, considering that 34.9% of teachers in this sample have suffered verbal abuse at least once, it was expected that the number of episodes of violence and complaints about professional performance would also show correlations with the dimensions of burnout, as can be seen in Table 5. Verbal victimization or threats are associated with burnout in a highly significant manner. On the other hand, having been the object of complaints did not show a relationship with the dimensions of burnout, but the number of complaints did show a statistically significant correlation, both with exhaustion and with cynicism.

### Multiple linear regression analysis

The relationships between burnout with the mentioned variables were analyzed using multiple linear regression analysis, taking into account each of the burnout dimensions, separately, as dependent variables. In this analysis, no statistically significant relationship was found between the dimensions of burnout with any demographic variable (age, gender, years of experience, etc.). No relationships were found between the dimensions of burnout and the environment (rural or urban) or the type of center (state or private).

It was confirmed that workload was the main variable when explaining exhaustion, in addition to community (interpersonal relationships in general)-, together with feelings of rejection, physical aggression, and insults. The model accounts for 56.4% of the global variance (Table 6).

**TABLE 6:** Linear regression model, taking as dependent variables: Exhaustion, Cynicism, and Efficacy.

Exhaustion	Coefficient	*P*-value	Confidence interval 95%	
			Lower	Upper
Constant	1.462	0.147	–3.193	21.125
Workload	2.037	<0.001	0.008	0.616
Community	2.541	<0.001	0.095	0.768
Rejection	2.876	0.005	0.152	0.829
Physical aggression	–0.387	0.026	–1.921	0.340
Insults	1.503	0.035	–0.260	1.892
R2	0.570			
R2adj	0.564			

**Cynicism**	**Coefficient**	***P*-value**	**Confidence interval 95%**	
			**Lower**	**Upper**

Constant	1.454	0.149	–2.821	18.329
Exhaustion	2.153	<0.001	0.014	0.344
Rewards	–3.224	<0.001	–1.038	–0.247
Fears	1.553	<0.001	–0.057	0.467
Rejection	0.505	0.042	–0.271	0.751
Insults	0.670	0.028	–0.932	0.941
Threats	–2.402	0.018	–2.070	–0.198
Claims	2.970	<0.001	0.437	2.191
R2	0.493			
R2adj	0.487			

**Efficacy**	**Coefficient**	***P*-value**	**Confidence interval 95%**	
			**Lower**	**Upper**

Constant	2.074	0.041	0.543	24.222
Exhaustion	3.986	<0.001	0.189	0.563
Cynicism	–3.030	<0.001	–0.540	–0.113
Workload	–2.635	<0.001	–0.690	–0.097
Rewards	2.388	0.019	0.094	1.012
Values	2.486	0.015	0.111	0.989
Fears	–2.956	0.004	–0.732	–0.144
Rejection	–1.392	<0.001	–0.173	0.497
Threats	2.770	<0.001	1.273	3.451
R2	0.364			
R2adj	0.355			

The variables that contributed to explaining the cynicism dimension were the exhaustion dimension, along with rewards for work, fears and rejection variables, and the number of episodes of insults, threats, and claims. This model explained 48.7% of the variance.

Finally, the degree of efficacy/inefficiency was explained in 35.5% by the other two dimensions of burnout, exhaustion and cynicism, as well as by the variables of workload, rewards, values, fears, rejection, and threats.

## Discussion

The results confirm the findings of other studies: individual characteristics, such as gender, age, or seniority, did not contribute to explain the development of burnout, but, rather, those variables related to working life (Maslach and Leiter, 2008). Similarly, no differences were found between burnout levels in different settings (urban or rural) or professionals working in state or private schools. Seniority only has an effect in the group for those with 3–6 years of experience, and affected only the dimension of emotional exhaustion.

Although the social support variable was not analyzed, the fact of living with a partner showed a statistically significant relationship with the dimension of cynicism. This result coincides with the majority of the research in which the relationships between perceived social support on stress and health have been analyzed (Ahola et al., 2006; Jakobsen et al., 2022).

It is impossible to describe every aspect of the work environment with which the person interacts. Especially in the educational environment. As in other studies, the weight that workload has on exhaustion has been verified, in the same way that this dimension maintains a high relationship with that of cynicism, and both in turn in the configuration of the feeling of efficacy or inefficiency in the job.

Also the aspects that make up the variables of working life, contribute differently in each profession. In secondary education, in addition to overload, the community area helps to explain the levels of exhaustion. The area of insufficient intrinsic rewards helps explain cynicism, while overload, lack of rewards, and value conflict explain, in part, levels of inefficacy.

Finally, the imbalance in the teacher-student relationship and the aggressions are related to the levels of the three dimensions that make up the burnout syndrome. Specifically, feelings of rejection, physical aggression, and insults show a statistically significant relationship with exhaustion. Feelings of fear and rejection of students, as well as threats and claims help explain the dimension of cynicism; and likewise, the fears a rejection, together with the episodes of threats that help explain the dimension of inefficiency.

Although due to the type of study and analysis, we cannot establish causal relationships, but rather relational ones. For example, the fact of having feelings of fear toward the students, or feeling rejection toward them or their behavior, could contribute to the dimension of exhaustion or cynicism. But, equally, teachers who felt tired and show responses of cynicism could facilitate mistrust, rejection, even violent situations between students and teachers.

Teacher-student relationships, and, in its most problematic aspect, aggressions toward educators by students or their families seem to be of great importance (Aloe et al., 2014; Krause and Smith, 2022).

It should be noted that there was no specific instrument to assess the degree of adjustment or maladjustment in the relationship of these professionals with their students. In this sense, the FREQ questionnaire, created and validated for this occasion, could be useful, showing good psychometric properties in two measurement moments and confirming a structure of two factors: fears and rejection.

Research on violence toward teachers has been abundant in the United States, but not so much in the rest of the world until recent years (Alzyoud et al., 2016; Galdames and Pezoa, 2016). Galand et al. (2007) tested through structural equation modeling–two models of the relationships between perceived school support, exposure to school violence, subjective wellbeing, and professional disengagement. Although the study only provides correlational information, the results reveal that school support is related to exposure to school violence, subjective wellbeing and professional disengagement, whereas the variable wellbeing moderates the effect of school violence on disengagement. However, the aforementioned study does not provide any measure of burnout, using instead a questionnaire for depression, anxiety, and somatization symptoms. A factor related to violence studied in this research is to have been a victim or witness of violence or to have received previous complaints from the pupils and/or parents. A few studies that have been published on this subject make it clear that being a victim or witness of violence is strongly related to anxious, depressive, or somatic symptoms (Galand et al., 2007).

Violence in the educational environment is a worrying issue for governments. Both that related to cases of bullying among the students themselves, and that exercised by teachers (Pişkin et al., 2014) and, as is the case in this study, the aggressive behavior of students or family members toward teachers (McMahon et al., 2017; Longobardi et al., 2019).

Much remains to be investigated, both on the variables involved in teacher burnout, and on the interactions between them, as well as the possible consequences of harmful–even aggressive–relationships on the mental and physical health of educators. This study confirms that variables such as the adjustment in the teaching relationship and the episodes of violence have a greater explanatory power on the burnout of secondary school teachers.

## Limitations

As a limitation is the design of the study as cross sectional studies cannot be understood as indicating causal relationships. Future studies should include structural equation modeling to examine the interrelation between burnout dimensions and violence-related variables.

Another limitation is, when the variables were conceptualized, the contextual variable “underprivileged environment” was not taken into account, whereas, as demonstrated in previous studies (Vercambre et al., 2009), working in an underprivileged context was associated with higher scores for both emotional exhaustion and cynicism, or depersonalization. On the other hand, we don’t include all contextual factors that have demonstrated high explanatory power in previous studies, such as number of pupils or teaching hours. Although the sample we based the study on is representative with regards to distribution among the two school types (state and private), the general conclusion about age or gender or school environment (urban or rural) have to be interpreted with caution. As justification for this, we considered that the Spanish educational system is highly homogeneous in this aspect, without striking differences in the number of teaching hours or number of pupils.

## Conclusion

It is worrying to note that one out of every three teachers have suffered verbal abuse at least once, and 3.8% have been a victim of physical abuse. Although physical violence is extremely rare (and low intensity), verbal victimization or threats are associated with burnout in a highly significant manner, which confirms previous findings about violence and burnout (Gascón et al., 2013a). Regarding the greater prevalence of verbal abuse, we must mention the work on incivility that has focused on the connections of bad social behavior with burnout and other forms of distress at work (Cortina et al., 2017).

The deterioration of teachers’ wellbeing and health is not only a personal harm, but also an attack on a common good and on the future development of the society that suffers them (Ervasti et al., 2012). Moreover, being educated in a climate of violence can lead to negative consequences during life (Maja et al., 2013). For this reason, there is a warning of the risk that may be posed by the fact that the classrooms in which the youngest are educated are dominated by a climate of fear or violence (Munn et al., 2007).

Although the methodology of this study does not allow establishing causal relationships, the data point to a relationship between physical and verbal aggression and other burnout variables; and should be used to design specific programs to equip teachers with the tools and skills necessary to cope with such abuse.

The Administration, which is pushing for teachers to be more resolute and innovative, does not provide the necessary means to do so. Although the Spanish government makes it understood that teachers are responsible for what happens in the classroom, it should protect teachers more from violence by creating new policies.

There is no room for doubt that burnout is a very complex syndrome and therefore requires treatment that will cover its multi-dimensionality. An effective intervention should address both the elements of the individual and organizational intervention (Awa et al., 2010), and the latter should incorporate protective measures against violence. Specifically, behavior modification programs should be incorporated and aimed at the management of stressful situations between the teacher and student in order to improve the relationship between both parties.

Taking into account the peculiarities of each educational system, these results could serve as a reference for other countries.

## Data availability statement

The original contributions presented in the study are included in the article/supplementary material, further inquiries can be directed to the corresponding author.

## Ethics statement

The studies involving human participants were reviewed and approved by Comisión de Ética en la Investigación de la Comunidad de Aragón. The patients/participants provided their written informed consent to participate in this study.

## Author contributions

BM, SG-S, MA, and RM-B: conceptualization. BM: data curation. BM, BO-B, and SG-S: formal analysis. BM, RM-B, and CB-M: investigation. BM, BO-B, SG-S, and RM-B: methodology. SG-S, AA, MA, and CB-M: project administration. AA and CB-M: resources. SG-S, MA, and AA: software. RM-B and SG-S: supervision and writing—original draft. BM and SG-S: validation. BM and BO-B: visualization. All authors contributed to writing—review and editing, contributed to the article, and approved the submitted version.

## References

[B1] Ahola K. Honkonen T. Isometsä E. Kalimo R. Nykyri E. Koskinen S. (2006). Burnout in the general population. *Soc. Psychiatry Psychiatr. Epidemiol.* 41 11–17. 10.1007/s00127-005-0011-5 16341826

[B2] Aloe A. M. Shisler S. M. Norris B. D. Nickerson A. B. Rinker T. W. (2014). A multivariate meta-analysis of student misbehavior and teacher burnout. *Educ. Res. Rev.* 12 30–44. 10.1016/j.edurev.2014.05.003

[B3] Alzyoud M. S. Al-Ali A. S. Tareef A. O. B. (2016). Violence against teachers in Jordanian schools. *Eur. Sci. J.* 12 1857–7881. 10.19044/esj.2016.v12n10p223

[B4] American Psychiatric Association (2013). *Diagnostic and statistical manual of mental disorders*, 5th Edn. Arlington, TX: American Psychiatric Publiishing. 10.1176/appi.books.9780890425596

[B5] Awa W. L. Plaumann M. Walter U. (2010). Burnout prevention: A review of intervention programs. *Patient Educ. Couns.* 78 184–190. 10.1016/j.pec.2009.04.008 19467822

[B6] Bakker A. B. Schaufeli W. B. Sixma H. J. Bosveld W. Van Dierendonck D. (2000). Patient demands, lack of reciprocity, and burnout: A five-year longitudinal study among general practitioners. *J. Organ. Behav.* 21 425–441. 10.1002/(SICI)1099-1379(200006)21:4<425::AID-JOB21>3.0.CO;2-#

[B7] Berg J. K. Cornell D. (2016). Authoritative school climate, aggression toward teachers, and teacher distress in middle school. *Sch. Psychol. Q.* 31 122–139. 10.1037/spq0000132 26524423

[B8] Bianchi R. Boffy C. Hingray C. Truchot D. Laurent E. (2013). Comparative symptomatology of burnout and depression. *J. Health Psychol.* 18 782–787. 10.1177/1359105313481079 23520355

[B9] Botía A. B. (2004). La educación secundaria obligatoria en España: En la búsqueda de una inestable identidad. *Rev. Iberoam. Sobre Calidad Eficacia y Cambio Educ.* 2:5.

[B10] Bresó E. Salanova M. Schaufeli W. B. Nogareda C. (2007). Síndrome de estar quemado por el trabajo “Burnout”(III): Instrumento de medición. I.N.S.H.T. *Nota Técn. Prev.* 732 1–4.

[B11] Burke R. J. Greenglass E. (1995). Responses and psychological burnout among teachers. *Int. J. Stress Manag.* 2 45–57. 10.1007/BF01701950

[B12] California Division of Occupational Safety and Health Administration. (1995). *Guidelines for workplace security. Department of industrial relations, division of occupational safety and health.* San Francisco, CA: California Division of Occupational Safety and Health Administration.

[B13] Claessens L. C. A. van Tartwijk J. van der Want A. C. Pennings H. J. M. Verloop N. den Brok P. J. (2017). Positive teacher – student relationships go beyond the classroom, problematic ones stay inside. *J. Educ. Res. Taylor Francis* 110 478–493. 10.1080/00220671.2015.1129595

[B14] Corbin C. M. Alamos P. Lowenstein A. E. Downer J. T. Brown J. L. (2019). The role of teacher-student relationships in predicting teachers’ personal accomplishment and emotional exhaustion. *J. Sch. Psychol.* 77 1–12. 10.1016/j.jsp.2019.10.001 31837719

[B15] Cortina L. M. Kabat-Farr D. Magley V. J. Nelson K. (2017). Researching rudeness: The past, present, and future of the science of incivility. *J. Occup. Health Psychol.* 22 299–313. 10.1037/ocp0000089

[B16] Donahue-Keegan D. Villegas-Reimers E. Cressey J. M. (2019). Integrating social-emotional learning and culturally responsive teaching in teacher education preparation programs. *Teach. Educ. Q.* 46 150–168.

[B17] Ervasti J. Kivimäki M. Pentti J. Salmi V. Suominen S. Vahtera J. (2012). Work-related violence, lifestyle, and health among special education teachers working in Finnish basic education. *J. Sch. Health* 82 336–343. 10.1111/j.1746-1561.2012.00707.x 22671950

[B18] Gadella Kamstra L. (2020). *Analysis of EFL teachers’ (de)motivation and awareness in spain doctoral dissertation.* Colchester: University of Essex.

[B19] Galand B. Lecocq C. Philippot P. (2007). School violence and teacher professional disengagement. *Br. J. Educ. Psychol.* 77 465–477. 10.1348/000709906X114571 17504557

[B20] Galdames A. M. Pezoa C. (2016). Violence towards secondary school teachers in the metropolitan region of chile. Rumbos TS. *Un Espacio Crítico Para Reflexión* 14 155–172.

[B21] Gascón S. Martínez-Jarreta B. González-Andrade J. F. Santed M. A. Casalod Y. Rueda M. A. (2009). Aggression towards health care workers in Spain: A multi-facility study to evaluate the distribution of growing violence among professionals, health facilities and departments. *Int. J. Occup. Environ. Health* 15 29–35. 10.1179/oeh.2009.15.1.29 19267124

[B22] Gascón S. Leiter M. P. Andrés E. Santed M. A. Pereira J. P. Cunha M. J. (2013a). The role of aggressions suffered by healthcare workers as predictors of burnout. *J. clin. Nurs.* 22 3120–3129. 10.1111/j.1365-2702.2012.04255.x 22978353

[B23] Gascón S. Leiter M. P. Stright N. Santed M. A. Montero-Marín J. Andrés E. (2013b). A factor confirmation and convergent validity of the “areas of worklife scale”(AWS) to Spanish translation. *Health Qual. Life Outcomes* 2013:63. 10.1186/1477-7525-11-63 23596987 PMC3637316

[B24] Hakanen J. J. Bakker A. B. Schaufeli W. B. (2006). Burnout and work engagement among teachers. *J. Sch. Psychol.* 43 495–513. 10.1016/j.jsp.2005.11.001

[B25] Instituto Aragonés de Estadística (2021). Available online at: https://opendata.aragon.es/datos/catalogo/estadisticas/03 (accessed September 21, 2022).

[B26] Jakobsen A. L. Hansen C. D. Andersen J. H. (2022). The association between perceived social support in adolescence and positive mental health outcomes in early adulthood: A prospective cohort study. *Scand. J. Public Health* 50 404–411. 10.1177/1403494821993718 33645305

[B27] Ju C. Lan J. Li Y. Feng W. You X. (2015). The mediating role of workplace social support on the relationship between trait emotional intelligence and teacher burnout. *Teach. Teach. Educ.* 51 58–67. 10.1016/j.tate.2015.06.001 31920801

[B28] Kakiashvili T. Leszek J. Rutkowski K. (2013). The medical perspective on burnout. *Int. J. Occup. Med. Environ. Health* 26 401–412. 10.2478/s13382-013-0093-3 24018996

[B29] Kowalczuk K. Krajewska-Kułak E. Sobolewski M. (2020). Working excessively and burnout among nurses in the context of sick leaves. *Front. Psychol.* 11:285. 10.3389/fpsyg.2020.00285 32158416 PMC7052176

[B30] Krause A. Smith J. D. (2022). Peer aggression and conflictual teacher-student relationships: A meta-analysis. *Sch. Ment. Health* 14 306–327. 10.1007/s12310-021-09483-1

[B31] Leiter M. P. Maslach C. (2003). Areas of worklife: A structured approach to organizational predictors of job burnout. *Res. Occup. Stress Well Being* 3 91–134. 10.1016/S1479-3555(03)03003-8

[B32] Longobardi C. Badenes-Ribera L. Fabris M. Martinez A. McMahon S. D. (2019). Prevalence of student violence against teachers: A meta-analysis. *Psychol. Violence* 9:596. 10.1037/vio0000202 35654486

[B33] Loonstra B. Brouwers A. Tomic W. (2009). Feelings of existential fulfilment and burnout among secondary school teachers. *Teach. Teach. Educ.* 25 752–757. 10.1016/j.tate.2009.01.002

[B34] Maja L. Siniša O. Vesna B. (2013). Violence against teachers: Rule or exception? *Int. J. Cogn. Res. Sci. Eng. Educ.* 1 6–15.

[B35] Maslach C. Jackson S. E. Leiter M. P. (1997). “Maslach burnout inventory,” in *Evaluating stress: A book of resources*, 3rd Edn, eds Zalaquett C. P. Wood R. J. (Lanham, MD: Scarecrow Education), 191–218.

[B36] Maslach C. Leiter M. P. (2008). *The truth about burnout: How organizations cause personal stress and what to do about it.* Hoboken, NJ: John Wiley & Sons.

[B37] McMahon S. D. Martinez A. Reddy L. A. Espelage D. L. Anderman E. M. (2017). “Predicting and reducing aggression and violence toward teachers: Extent of the problem and why it matters,” in *The Wiley handbook of violence and aggression Volume 3. Societal interventions*, ed. Sturmey P. (Hoboken, NJ: John Wiley & Sons), 1335–1350. 10.1002/9781119057574.whbva100

[B38] Montero-Marín J. Prado-Abril J. Carrasco J. M. Asensio Á Gascón S. García-Campayo J. (2013). Causes of discomfort in the academic workplace and their associations with the different burnout types: A mixed-methodology study. *BMC Public Health* 13 1–14. 10.1186/1471-2458-13-1240 24377904 PMC3878796

[B39] Munn P. Johnstone M. Sharp S. Brown J. (2007). Violence in schools: Perceptions of secondary teachers and head teachers over time. *Int. J. Violence Sch.* 3 52–80.

[B40] Pietarinen J. Pyhältö K. Soini T. Salmela-Aro K. (2013). Reducing teacher burnout: A socio-contextual approach. *Teach. Educ.* 35 62–72. 10.1016/j.tate.2013.05.003

[B41] Pişkin M. Gökhan A. T. Çinkir Ş Öğülmüş S. Babadoğan C. Çokluk Ö (2014). The development and validation of the teacher violence scale. *Eurasian J. Educ. Res.* 56 1–20. 10.14689/ejer.2014.56.3

[B42] Rahman M. S. (2020). The advantages and disadvantages of using qualitative and quantitative approaches and methods in language “testing and assessment” research: A literature review. *J. Educ. Learn.* 6 102–112. 10.5539/jel.v6n1p102

[B43] Reed G. M. First M. B. Kogan C. S. Hyman S. E. Gureje O. Gaebel W. (2019). Innovations and changes in the ICD-11 classification of mental, behavioural and neurodevelopmental disorders. *World Psychiatry* 18 3–19. 10.1002/wps.20611 30600616 PMC6313247

[B44] Riolli L. Savicki V. (2006). Impact of fairness, leadership, and coping on strain, burnout, and turnover in organizational change. *Int. J. Stress Manag.* 13:351. 10.1037/1072-5245.13.3.351

[B45] Salanova M. Schaufeli W. (2000). Desde el “Burnout” al “Engagement”: ¿una nueva perspectiva? *J. Work Organ. Psychol.* 16 117–134.

[B46] Schaufeli W. B. Leiter M. P. Maslach C. (2009). Burnout: 35 years of research and practice. *Career Dev. Int.* 14 204–220. 10.1108/13620430910966406

[B47] Skaalvik E. M. Skaalvik S. (2011). Teacher job satisfaction and motivation to leave the teaching profession: Relations with school context, feeling of belonging, and emotional exhaustion. *Teach. Teach. Educ.* 27 1029–1038. 10.1016/j.tate.2011.04.001

[B48] Smith D. L. Smith B. J. (2006). Perceptions of violence: The view of teachers who left urban schools. *High Sch. J.* 89 34–42. 10.1353/hsj.2006.0004 34409987

[B49] Teuber Z. Nussbeck F. W. Wild E. (2021). School burnout among Chinese high school students: The role of teacher-student relationships and personal resources. *Educ. Psychol.* 41 985–1002. 10.1080/01443410.2021.1917521

[B50] Tiesman H. M. Hendricks S. Konda S. Hartley D. (2014). Physical assaults among education workers: Findings from a statewide study. *J. Occup. Environ. Med.* 56 621–627. 10.1097/JOM.0000000000000147 24854254 PMC4570474

[B51] Toppinen-Tanner S. Ahola K. Koskinen A. Väänänen A. (2009). Burnout predicts hospitalization for mental and cardiovascular disorders: 10-year prospective results from industrial sector. *Stress Health* 25 287–296. 10.1002/smi.1282

[B52] Van Droogenbroeck F. Spruyt B. (2015). Do teachers have worse mental health? Review of the existing comparative research and results from the Belgian Health Interview Survey. *Teach. Teach. Educ.* 51 88–100. 10.1016/j.tate.2015.06.006

[B53] Vahtera J. Kivimäki M. Pentti J. Linna A. Virtanen M. Virtanen P. (2004). Organisational downsizing, sickness absence, and mortality: 10-town prospective cohort study. *BMJ* 328:555. 10.1136/bmj.37972.496262.0D 14980982 PMC381046

[B54] Vercambre M. N. Brosselin P. Gilbert F. Nerrière E. Kovess-Masféty V. (2009). Individual and contextual covariates of burnout: A cross-sectional nationwide study of French teachers. *BMC Public Health* 9:333. 10.1186/1471-2458-9-333 19744328 PMC2754990

[B55] von der Embse N. Ryan S. V. Gibbs T. Mankin A. (2019). Teacher stress interventions: A systematic review. *Psychol. Sch.* 56 1328–1343. 10.1002/pits.22279

[B56] Winstanley S. Whittington R. (2004). Aggression towards health care staff in a UK general hospital: Variation among professions. *J. Clin. Nurs.* 13 3–10. 10.1111/j.1365-2702.2004.00807.x 14687287

[B57] Zhao X. Ding S. (2019). Phenomenology of burnout syndrome and connection thereof with coping strategies and defense mechanisms among university professors. *Eur. J. Invest. Health Psychol. Educ.* 10 82–93. 10.3390/ejihpe10010008 34542471 PMC8314242

